# Changes in intra-host mycovirus population diversity after vertical and horizontal transmission

**DOI:** 10.1093/ve/veaf082

**Published:** 2025-10-23

**Authors:** Karla Peranić, Deborah M Leigh, Maja Popović, Lucija Nuskern, Mirna Ćurković-Perica, Quirin Kupper, Daniel Rigling, Marin Ježić

**Affiliations:** Division of Microbiology, Department of Biology, Faculty of Science, University of Zagreb, Marulićev trg 9a, 10000 Zagreb, Croatia; Senckenberg Nature Research, Genomic Biomonitoring, , Senckenberganlage 25, 60325 Frankfurt, Germany; Swiss Federal Institute for Forest, Snow and Landscape Research, WSL, Zürcherstrasse 111, 8903 Birmensdorf, Switzerland; Institute of Ecology, Evolution, and Diversity, Faculty of Biosciences, Goethe University Frankfurt, Max-von-Laue-Str. 9, 60438 Frankfurt, Germany; Institute of Forest Genetics, Dendrology and Botany, Faculty of Forestry and Wood Technology, University of Zagreb, Svetošimunska 23, 10000 Zagreb, Croatia; Division of Microbiology, Department of Biology, Faculty of Science, University of Zagreb, Marulićev trg 9a, 10000 Zagreb, Croatia; Division of Microbiology, Department of Biology, Faculty of Science, University of Zagreb, Marulićev trg 9a, 10000 Zagreb, Croatia; Swiss Federal Institute for Forest, Snow and Landscape Research, WSL, Zürcherstrasse 111, 8903 Birmensdorf, Switzerland; Swiss Federal Institute for Forest, Snow and Landscape Research, WSL, Zürcherstrasse 111, 8903 Birmensdorf, Switzerland; Division of Microbiology, Department of Biology, Faculty of Science, University of Zagreb, Marulićev trg 9a, 10000 Zagreb, Croatia

**Keywords:** chestnut blight, hypovirulence, virus evolution, PacBio, bottleneck effect

## Abstract

The remarkable speed at which viral populations mutate allows them to evolve quickly, so that the viral diversity can change, especially when the virus is transmitted, i.e. its population goes through a bottleneck. Our experiments assessed the diversity of the intra-host populations of a mycovirus Cryphonectria hypovirus 1 (CHV1), a natural biocontrol agent of chestnut blight disease, using PacBio long-read HiFi sequencing. The intra-host viral population diversity before and after either vertical or horizontal transmission was estimated using two metrics—nucleotide (mutational) diversity measured as π, and viral variant diversity measured as Nei’s H. A significant bottleneck effect, demonstrated by the decline of the mutational diversity (π), was observed after vertical transmission of prototypical viral populations into conidia, in both investigated viral subtypes, French 1 (F1) and Italian (I). In contrast, the number of viral variants was significantly reduced after the vertical transmission of subtype I but increased for the subtype F1. In newly isolated fungal strains infected with CHV1 subtype I, fewer viral variants were vertically transferred into conidia, relative to the prototypical laboratory isolates, i.e. the average number of transmitted viral variants was smaller. In the horizontal viral transmission assays, the number of transmitted viral variants was closely linked to the genotype of the fungal host at the vegetative compatibility loci. Specifically, recipient viral populations’ diversity was greater when the alleles at loci *vic2* and *vic3* were the same in the donor and recipient fungal isolate, relative to when they were different. Heteroallelism at the *vic4* locus had no impact on viral populations’ diversity*.* Despite the strong bottlenecks, purifying selection shaped the diversity of intra-host CHV1 populations. In both transmission experiments on average, synonymous mutational diversity was higher than non-synonymous, across all replicates. Signs of positive selection or mutation accumulations, inferred by a surplus of nonsynonymous mutations, were less common and mostly observed during vertical transmission experiments, i.e. in new viral populations arising from conidia.

## Introduction

RNA viruses are the most abundant parasites infecting animals and humans, domesticated plants, fungi, and bacteria (Domingo and Holland 1997) and are notable for their large census population size, short generation time, and high replication and mutation rates (Southwood and Ranganathan 2019), which leads to rapid generation of extremely high genetic variability (Domingo and Holland 1997). These characteristics also enable rapid adaptation of RNA viruses, enabling them to overcome host defences and develop resistance to drug treatments, primarily by generating new variants with altered biological properties (Gutiérrez et al. 2012). Much of our understanding of viruses stems from human pathogen systems, but wider taxonomic analysis of the genetic structure and evolution of viral populations is vital for the development of strategies for disease or epidemic control, as well as the development of strategies for sustainable use of RNA viruses, e.g. in biocontrol of plant diseases.

Analyses of the genetic structure and evolution of intra-host populations are crucial to the development of strategies for viral disease control (Jridi et al. 2006). It has been hypothesized that the intra-host viral genetic diversity is dominated by a few viral variants and that the rest of the population consists of variants found in lower frequency (Eliseev et al. 2020). A series of population bottlenecks during plant infection results in genetic drift of viral populations, while coinfection by more than one viral genotype results in competition for resources, all of which create selection pressure on the virus population (Frank 2001, Holmes 2009, García-Arenal and Fraile 2013, Pagán and García-Arenal 2018). Selection (specifically), or genetic bottleneck (randomly), can cause a reduction of the mutation frequency in a population. The frequency of the fittest variants in a specific environment will increase (positive selection) while the less fit variants will be eradicated from the population (negative selection). On the other hand, genetic bottlenecks are stochastic events that reduce the overall genetic variation of a population and can lead to genetic drift (Ali et al. 2006). Adaptation of a virus can be fast when natural selection dominates its evolution, while the random variation in allele frequencies that results when genetic drift is the predominant evolutionary force can alter the direction that would otherwise be driven by selection, slowing down the adaptation (Gutiérrez et al. 2012).

*Cryphonectria parasitica* is a phytopathogenic fungus from the phylum Ascomycota. Both sexual and asexual spores of *C. parasitica* can cause an infection of chestnut tree bark. The fungus is native to eastern Asia and spread to North America and Europe during the twentieth century through accidental introduction (Rigling and Prospero 2018). The fungus causes bark cankers on susceptible trees, a disease that primarily affects chestnut trees (*Castanea* spp.), with serious effects on American and European forest ecosystems. While the spread of this fungus throughout European chestnut populations caused considerable damage, by chance, a hyperparasitic mycovirus, Cryphonectria hypovirus 1 (CHV1), was co-introduced. The virus causes chronic fungal infections that attenuate the virulence of the host, a phenomenon called hypovirulence, mitigating the most severe damage to infected trees. As a result, CHV1 is now widely used for biocontrol across Europe.

CHV1 is a positive-sense single-stranded RNA virus, with a dsRNA replicative form that accumulates abundantly, but without a capsid protein (Fahima et al. 1993). CHV1 infection significantly alters the gene expression of *C. parasitica* genes associated with metabolism and virulence (Chun et al. 2020). As a result, it reduces fertility, i.e. sporulation and parasitic growth of the infected fungus, thus giving the chestnut tree time to heal and survive the infection (Anagnostakis 1982, Brusini et al. 2017, Rigling and Prospero 2018).

CHV1 is transmitted both vertically *via* asexual spores—conidia, and horizontally by hyphal anastomosis between different fungal individuals, during which a transmission bottleneck is expected to occur, i.e. different number of viral genomes can be transmitted from one host to another, establishing a new infection (Ghafari et al. 2020). The transmission rate into the conidia is dependent on the fungal and viral genotypes (Peever et al. 2000, Bryner and Rigling 2011), while the horizontal transmission is affected by a vegetative incompatibility system, which is common in many fungal species (Liu and Milgroom 1996, Cortesi et al. 2001). The system prevents the formation of stable anastomosis, i.e. the fusion of the fungal hyphae between incompatible fungal genotypes. As a result, the cytoplasmic exchange between individuals is restricted, so the system functions as a defence mechanism against pathogens such as mycoviruses, which can be transmitted *via* cytoplasm (Caten 1972). To date, six diallelic, unlinked, vegetative incompatibility (*vic*) loci have been identified (Cortesi and Milgroom 1998), defining 64 *vic* genotypes, which correspond to 64 different vegetative compatibility (vc) types (known in Europe as EU types). Two fungal individuals are considered compatible if they have the same alleles at all *vic* loci; therefore, virus transmission between them isn’t restricted. However, heteroallelism at particular *vic* loci may significantly reduce the efficiency of virus transmission (Liu and Milgroom 1996, Cortesi et al. 2001, Papazova-Anakieva et al. 2008). The consequence of this phenomenon is that the expected virus transmission, i.e. the average transmission rate predicted for any two randomly chosen individuals from a population, is negatively correlated with vc type diversity of the population (Liu and Milgroom 1996). Finally, sexual reproduction of the host fungus can limit virus spread throughout the population, because it can generate new allelic combinations at *vic* loci and also because the virus isn’t transmitted by sexual spores—ascospores (Anagnostakis 1988, Prospero et al. 2006). Therefore, the efficiency of vertical and horizontal virus transmission is a crucial factor in determining the potential for successful biological control (Ding et al. 2007).

Genomic research of rapidly evolving RNA viruses benefits greatly from next-generation sequencing (NGS) technologies because they enable the study of intra-host viral diversity. Of particular value are those that generate longer sequences with minimum intrinsic error rates because they allow direct examination of different intra-host viral variants (Satam et al. 2023). One such long-read sequencing technology is PacBio (Pacific Biosciences) sequencing or SMRT (single-molecule real-time) sequencing. It uses circular consensus sequencing (ccs), i.e. the DNA template is circularized, which allows multiple sequencing passes of the same molecule. The results are multiple reads of the same sequence from which a consensus can be constructed. This eliminates most of the sequencing errors because the errors that are randomly distributed along the read sequence can be easily identified and removed (Carneiro et al. 2012), thus greatly improving the read accuracy (Travers et al. 2010). In viruses with high mutation rates such as HIV-1, where the intra-host viral diversity is high, the quality of viral variant reconstruction was highly variable and poor with older NGS technologies (Eliseev et al. 2020). PacBio sequencing generates long reads with high consensus accuracy and uniform coverage (Kingan et al. 2019), thus allowing us to successfully detect intra-host viral variants, including the rare ones, which is of the utmost importance for diversity analysis (Hamim et al. 2022).

## Aims

The aims of this research were to: (i) explore the horizontal and vertical transmission rates of CHV1 from donor to recipient fungal strain or conidial progeny, (ii) investigate the mutational diversity and the variant diversity changes in viral populations of two CHV1 subtypes, French 1 (F1) and Italian (I), after vertical and horizontal transmission, (iii) determine the phylogenetic relationships of the viral populations before and after transmission, and (iv) explore the intra-host patterns of selection during the CHV1 transmission.

## Materials and methods

### Vertical transmission of CHV1 into conidia

Three hypovirus-infected *C. parasitica* isolates harbouring one previously well-characterized Croatian CHV1 strain, CR23 (subtype I), two prototypic CHV1 strains: EP713 (French 1 subtype, F1), Euro7 (Italian subtype, I), and three hypovirus-infected *C. parasitica* isolates sampled in 2019 in Croatia (Leigh et al. 2021): oz_08.2, ks_30.4, and ks_46.4 (all infected by CHV1 subtype I), were used in this study. Isolate EP713 was obtained by introducing CHV1 from a French strain into the hypovirus-free strain EP155 (Anagnostakis 1981), while Euro7 was isolated in 1978 from a superficial canker ~30 km north of Florence, Italy (Chen and Nuss 1999). Isolate CR23 was isolated in Croatia in one of the previous studies (Krstin et al. 2008). All hypovirus-infected fungal isolates were stored at −80°C in 22% glycerol and were regrown for this research by inoculation on a 90 mm-diameter Petri dish containing 20 ml of potato dextrose agar (PDA) (BD, Difco). The experiments were carried out in a climate chamber under controlled conditions: in the dark, at 24°C and 70% humidity, unless specified differently. When the growing colonies were ~5 cm in diameter, the tissue was divided into four parts and each part was transferred to a separate Petri dish with cellophane overlaid over one-half of the PDA medium. One-half growing directly on the PDA was used for the collection of conidia, and the other half growing on the cellophane was used for the collection of mycelia for other analyses. The colonies were grown for 2 weeks in a climate chamber to promote growth, and subsequently for four more days on a laboratory bench, exposed to daylight, to promote conidia formation. The asexual spores were collected by washing the colonies with 2 ml of sterile deionized water, gently scraping the surface, and subsequently carefully checking under a microscope to ensure no mycelia were accidentally harvested. The collected conidia were counted with a cell counting chamber (Neubauer-improved, Hirschmann), and the conidial suspensions were then diluted to contain approximately five conidia per 100 μl. For each sample, 500 μl of the diluted conidial suspension was plated onto five new 90 mm Petri dishes (100 μl on each plate) to ensure the growth of approximately five colonies per plate. After two to three days, when the new colonies were formed, they were divided in half and transferred to two new Petri dishes with PDA overlaid with cellophane to obtain enough mycelia for the subsequent analyses. Between 11 and 17 colonies derived from spores of each analysed hypovirus-infected fungal isolate were grown. RNA was isolated from the newly grown fungal isolates using a dsRNA isolation kit (iNtRON), and RT-PCR detection of the CHV1 was performed. Reverse transcription (cDNA synthesis) was performed using the GoScript™ Reverse Transcription System (Promega, Madison, WI, USA) according to the manufacturer’s recommendation, and PCR was performed on the obtained cDNA with Taq G2 polymerase (Promega, Madison, WI, USA) as described by Allemann et al. (1999). The obtained amplicons were separated on 1% (w/v) agarose gel in 0.5× TBE buffer at 5 V/cm and visualized with GelStar DNA stain (Lonza, Basel, Switzerland). For all the single spores that tested positive for CHV1, the mycelia that had been collected from the cellophane were lyophilized and ground to a fine powder with φ 5 mm steel ball in TissueLyser II (Qiagen, Venlo, Netherlands) for 2 min at 30 Hz. The ground mycelia were then used for subsequent analyses. The success of the vertical transmission of the CHV1 was expressed as percentage, and the differences between the transmission success rates among virus strains were calculated using Fisher’s exact test implemented in Past 4.03.

### Horizontal transmission of CHV1 between different vegetative compatibility types

The effect of the vegetative incompatibility on horizontal virus transfer was analysed utilizing three hypovirus-infected fungal isolates as viral donors: Euro7 donor (ED; EU-9), EP713 donor (FD; EU-5), and CR23 donor (CD; EU-1), as well as several recipient hypovirus-free isolates. We focused on heteroallelism at three out of six known *vic* loci: *vic2* (strong restriction of CHV1 transfer), *vic3* (intermediate restriction), and *vic4* (low restriction). Thus, each of the donors: Euro7, EP713, and CR23, was paired with three vc type tester isolates (or field isolate HK22C in one instance), one for each *vic2, vic3*, and *vic4* difference. Specifically, Euro7 was paired with EU-15, EU-36, and EU17; EP713 with EU-6, EU-60, and EU-1; and CR23 with HK22C (=EU-2), EU-44 and EU-5. Tester strains EU-15, EU-6, and field isolate HK22C were used to test the effect of *vic2* difference, EU-36, EU-60, and EU-44 to test the effect of *vic3* difference, and EU-17, EU-1, and EU-5 to test the effect of *vic4* difference, respectively (Supplementary Table S1). Virus-free, field isolate HK22C, genotyped in a previous study (Ježić et al. 2018), was used because no successful transmissions occurred into the EU-2 tester isolate. Each of these pairings was performed in at least 25 replicates. As a control, we analysed the pairings of the donor fungal isolates with vegetatively compatible standard EU tester isolates, i.e. both the donor and the recipient had the same *vic* genotype. Specifically, Euro7, EP713, and CR23 were paired with EU-9, EU-5, and EU-1, respectively. These pairings were done in ten replicates.

In order to increase the repeatability of the procedures and reduce unaccounted biases, all donor and recipient fungal isolates were taken from our long-term storage stocks kept at −80°C in 22% glycerol, and grown on a 60 mm-diameter Petri dish with PDA for 5 days in a growth chamber at the aforementioned conditions. To induce horizontal transfer of the CHV1, a small, approximately 3x3x3 mm cube of freshly grown mycelia of the donor fungal isolate was placed near a similarly sized cube of the recipient fungal isolate close to the margin of a Petri dish (Liu and Milgroom 1996). The pairings were monitored daily to assess if the transmission of CHV1 had occurred. After ~14 days, the mycelia of the recipient fungus with changed morphology, i.e. converted to white, hypovirus-infected morphology, were cut into four pieces and placed on four new 90 mm-diameter Petri dishes with fresh PDA, overlaid with cellophane. The same procedure was applied to the mycelia of the hypovirus-infected donor as well. After 10 days of growth in the growth chamber, CHV1 presence was assessed in the same manner as in the vertical transmission experiment to confirm successful virus transmission. To confirm that we have indeed taken only recipient tissue from the PDA plate after transformation, we have isolated fungal DNA from the recipient tissue as well. DNA isolation was performed with Omniprep for fungus kit (G bioscience), followed by PCR using Taq G2 polymerase (Promega). Genotyping of *vic* loci distinguishing donor and recipient was done using protocols described in Mlinarec et al. (2018). After the virus transmission was confirmed, the collected mycelia of all donor and recipient fungal isolates were lyophilized, ground, and stored, as described above, for further analyses. The donor fungal isolates, CR23, Euro7, and EP713, were also grown in the same conditions as described above, but alone, without being involved in the CHV1 transmission experiment, to determine the potential difference in the viral population diversity; these were named: pooled.CR23 (CP), pooled.Euro7 (EP), and pooled.EP713 (FP).

### Library preparation for PacBio sequencing

Library preparation and further bioinformatics analyses were done as described in Leigh et al. 2021 with minor alterations. To isolate RNA, the replicative form of CHV1 (dsRNA) was isolated using a dsRNA extraction mini kit (iNtRON) from ~30 mg of lyophilized mycelia following the manufacturer’s protocol. The concentration of total RNA was measured using Qubit (BR, Thermo Fischer, USA). The extracted dsRNA was incubated for 2 min at 100°C and then chilled on ice. Maxima H Minus Reverse Transcriptase (Thermo Scientific) and oligo(dT)_12–18_ primers (the plus-strand RNA of CHV1 has a poly(A) tail) were used to synthesize cDNA per the manufacturer’s instructions. Long-range PCR was performed immediately to diminish the possibility of heteroduplex formation during the PCR (Waugh et al. 2015). The target area of the CHV1 genome was a 5 kb amplicon obtained utilizing the primer pair ATCYGGAGAARGTGATTTGC and YTTRTTGATGTAGCTGCGAGG. A 6 bp barcode was used to tag the primers so that the particular samples could be easily identified (barcode sequences supplied by B. Murrell). The selected part of the CHV1 genome spans around 40% of its 12.7 kb genome from the 5′-end, and it consists of a noncoding region, the ORFA that encodes a papain-like protease p29, and a highly basic protein p40, derived from polyprotein p69, by a p29-mediated cleavage event, and a part of ORFB that contains a second papain-like protease p48. The rest of the ORFB—a putative RNA polymerase and helicase domains are not within our 5 kb amplicon (Shapira et al. 1991). The PCR cycling conditions were: initial denaturation for one minute at 95°C, followed by 15 cycles consisting of two steps: 30 s at 95°C and 7 min at 68°C, ending with the final extension for ten minutes at 68°C. The PCR protocol was repeated five times for each sample, i.e. viral populations from a particular experiment, using high fidelity Advantage 2 Taq Polymerase (Takara, Japan), which were then pooled and purified using Ampure beads (Beckman Coulter, USA) and the DNA concentrations were once again measured using Qubit. The barcoded samples were equimolarly pooled and the pool spread across PacBio Sequel SMRTcells. Further PacBio library preparation steps and sequencing were done at the Functional Genomics Centre Zurich (Switzerland).

### Long-read processing of the obtained raw sequence data

Before sequencing, hairpin adapters were ligated to the amplicons obtained in Library Preparation for PacBio Sequencing in order to circularize linear DNA and enable the polymerase to make multiple passes of resequencing of the same amplicon. The obtained reads were collapsed into so-called ‘Circular Consensus Sequences’ (CCS) or HiFi reads with *pbccs* software (Pacific Biosciences, California) that have higher accuracy and reduced sequencing error. From these multiple reads, a single long, high-quality HiFi read is produced. For reads to be included in further analysis, certain conditions had to be met: minimum length of 3 kb, 5 polymerase passes, and a predicted sequencing quality of 0.99. *Lima* (Pacific Biosciences, California) was used as a demultiplexing tool, more specifically, viral variants from a particular sample, i.e. viral population composition from a particular experiment, were identified using the barcodes attached to the primers. Barcodes were allowed a minimum quality score of 26, as well as different forward and reverse barcodes to match our read structure.

### Mutation detection

The demultiplexed files in ‘bam’ format were converted to a ‘fasta’ format using *samtools* (v1.13, Li et al. 2009). The converted files were aligned to a CHV1 reference sequence: for subtype I strain EP721, accession number DQ861913 (Lin et al. 2007) and for subtype F1 strain EP713, accession number NC_001492 (Shapira et al. 1991), using *minimap2* (v2*.*19, Li 2016) with the ‘map-pb’ option suitable for PacBio reads. These reference sequences were chosen to ensure cross-comparability with other CHV1-based studies (*e.g.*  Bryner et al. 2012, Feau et al. 2014, Murolo et al. 2018, Leigh et al. 2021, Ahmad et al. 2024). The resulting files were sorted, indexed, and converted to a ‘bam’ format with *samtools.* Then the intra-host mutations were called utilizing two programs: *Freebayes* (v.1.3.1, Garrison and Marth 2012) and *Deepvariant* (v.1.1.0, Poplin et al. 2018) using the PacBio option and assuming a ploidy 1 with ‘pooled discrete’ and ‘pooled continuous’ option activated. Mutational diversity (π) was used as a measurement for intra-host viral population diversity and calculated using SNPGenie software (Nelson et al. 2015) with a custom annotation of genomic structure developed for CHV1 using published descriptions (reviewed in Nuss (2005)). Since SNPGenie reports genome-wide values of π, the values were corrected to the amplicon region by calculating the average sum of pairwise differences at all coding sites in the amplicon, divided by the total number of sites (Ndiffs+Sdiffs/NSites+SSites in the SNPGenie codon file). No minimum allele frequency or sliding window was used. The viral population intra-host diversity was measured using π because it is robust to large variations in sequencing depth (Zhao and Illingworth 2019). Mutation effects were also annotated using SNPEff (v4.3, Cingolani et al. 2012) and a custom genome annotation for CHV1. Mutational effects were defined following SNPEff’s standard approach (Cingolani et al. 2012) and specifically following the SNPEff manual.

### Viral variant detection

As there is always a certain sequencing error, PacBio HiFi reads had to be denoised and merged in order to generate accurate CHV1 variants. Quality-filtered and demultiplexed reads were run through the Robust Amplicon Denoising pipeline specifically designed for PacBio HiFi reads (Kumar et al. 2019). After that, the files were converted to ‘fasta’ format and run through the programming language R using the package *pegas* (v0.14, Paradis 2010) to calculate the Hamming distance between them (Bierbrauer 2016). The Hamming distance, in our case, refers to the number of positions in which two sequences of the same length differ. Mathematically, for strings *a* and *b* of length *n*, the Hamming distance *dH*(*a*, *b*) can be expressed as:


$$ dH\left(a,b\right)={\sum}_{i=1}^n\left( ai\ne bi\right) $$

where *n* is the length of the strings, *ai* and *bi* are the symbols at the *i*-th position of strings *a* and *b*, respectively, and (*ai* ≠ *bi*) is a conditional expression that evaluates to 1 if *ai* and *bi* are different, and 0 otherwise. The read count of all the sequences that were the same, i.e. the Hamming distance between them was 0, was combined to calculate the correct number of a particular viral variant detected. The diversity of detected viral variants of each analysed viral population was calculated with Nei’s H (gene diversity measurement, Nei and Tajima 1981). The formula used is:


$$ h=n\left(1-{\sum}_{i=1}^l{x}_i^2\right)\big/\left(n-1\right) $$

where *n* is the variant number and *X_i_*^2^ is the squared frequency of the variants.

Before the alignment with *minimap2*, the CHV1 viral variants obtained from parental viral populations and their respective conidial progeny, as well as donor viral populations and corresponding recipient populations, were merged and run through R utilizing the *pegas* package, using the Hamming distance to identify which viral variants are shared between them.

The average number of mutations, i.e. the average number of all of the mutations (SNPs) observed at a certain genome position in a viral population, and the average number of viral variants (unique haplotypes) for a parental and progeny viral populations were calculated from the number of mutations and viral variants from each viral population (Table 2).

### Viral population diversity

A general linear model (GLM) was used to test significant differences between groups of viral populations: experimental strains used in vertical virus transmission EP713, Euro7, and CR23 and the field isolates ks_30.4, ks_46.4, and oz_8.2 and their respective conidial progeny. We tested differences between frequencies of viral variants observed in parental and their particular progeny populations, as well as between parental and all their progeny populations combined. The same approach was used in the horizontal transfer experiment between viral donors’ populations and their respective recipients based on the vc-type difference, i.e. differences were tested between donor and recipient virus population for each individual transmission assay, as well as for the combined replicates of recipient populations for each heteroallelic locus. As GLM indicated significant differences only in one case of vertical transfer—between populations of ks_46.4 parental populations and their conidial progeny, *post hoc* Tukey Least Significant Difference (LSD) test was used to test the differences between all pairs of populations in that particular experiment.

The differences in the diversity of viral populations, expressed either as mutational diversity index (π), or number of detected viral variants, were estimated between the parental viral populations and their conidial progeny, as well as between donor viral populations, recipient populations and pooled viral populations (i.e. not involved in transmission experiment), using a linear mixed-effects model (R version 4.0.5 using lme4 v1.1–35.1, Bates et al. 2015). To better fit the model, the variant number was used instead of Nei’s H and the mutational diversity and variant number were log-transformed. The multivariate analysis of variance (MANOVA) was used to see if there was a significant difference between the diversity of viral populations i.e. Nei’H and π, considering different factors: particular viral strain, viral subtype, and, in horizontal transmission, vc-type discordance involved in the experiment. For significant results, ANOVA was used to determine which diversity metric has a statistically significant difference. Finally, Fisher’s LSD *post hoc* test was performed to test which of the aforementioned groups differ using the *agricolae* package in R.

To determine if there were differences in the frequency of successful vertical viral transfers into the spores, Fisher's exact tests were performed in order to compare the number of hypovirus-free and hypovirus-infected isolates grown from spores that were produced by different parental fungal isolates, i.e. different parental viral populations. This was done in order to test different viral strains’ efficiency of vertical transmission. Similarly, the Fisher exact test was used to test the differences in the number of successful and unsuccessful horizontal viral transfers into the recipient fungal isolate, depending on the vc type difference between donor and recipient and the particular CHV1 strain involved in the experiment.

### Phylogenetic relationship reconstruction

Phylogenetic reconstruction of inter-host relationships between viral populations was inferred using RAxML-NG (v0.9.0, Kozlov et al. 2019). Viral populations for which phylogenetic reconstruction was performed were divided by the CHV1 subtype: F1 or I, and by the particular transmission experiment performed: vertical or horizontal. Host-specific consensus sequences were constructed using *bcftools* (v1.15, Li et al. 2009), which generates a consensus using the reference genome as a backbone and intra-host mutations. RAxML-NG was run with an ‘all-in-one’ analysis and a GTR + G model with 1000 bootstrap replicates. Transfer (TBE) bootstrap metrics were calculated, and the values are presented on the phylogenetic tree using iTOL (v.6.9, Letunic and Bork 2024).

Mutations in intra-host CHV1 viral populations were predicted by SNPGenie (Nelson et al. 2015) and SNPEff (Cingolani et al. 2012) to be either synonymous or non-synonymous. The intra-host mutational diversity was measured using nucleotide diversity, i.e. π, which was used to predict intra-host patterns of selection by comparing π of nonsynonymous and synonymous mutations. The comparison was done separately for different types of transmission, i.e. vertical or horizontal. We also explored whether there was a difference in intra-host patterns of selection between parental populations and conidial progeny, as well as between donor populations and recipient viral populations. Furthermore, the comparison was done for the whole 5 kb amplicon but also for the specific part of the CHV1 genome, either ORFA or ORFB.

## Results

A total of 88 samples, i.e. viral populations involved in the experiments, were sequenced: (1a) hypovirus-infected parental populations from vertical transmission experiments: EP713 (FM), Euro7 (EM), CR23 (CM), oz_08.2. (OM), ks_30.4 (K1M) and ks_46.4 (K2M) and (1b) 4–6 viral populations from conidial progeny derived from each of the aforementioned parental population; 2a) hypovirus-infected donor viral populations: EP713 (FD), Euro7 (ED), and CR23 (CD) that were either cocultured with a particular EU tester involved in horizontal CHV1 transmission (referred to as ‘donor’) or (2b) grown on their own (referred to as ‘pooled’), and (2c) three replicates of newly established viral populations after the transmission (referred to as ‘recipients’) (Table 1.)

**Table 1:** List of analysed viral populations.

Population name abbreviation	Sample and replicate names
FM	EP713 parental isolate in the vertical transmission (mother.EP713)
FS1-FS6	EP713 spores’ viral populations (one to six)
Spore.EP713	All of the EP713 spores’ viral populations combined
FP	Pooled.EP713 sample i.e. EP713 mycelia not part of any transmission experiments representing a ‘baseline’ viral diversity of that sample
FD5, −6, −60, −1	EP713 donor isolate (EU-5) paired with EU-5, EU-6, EU-60, EU-1 tester respectively
Donor.EP713	All of the EP713 donors’ viral populations combined
F51-F53, 61–63, 601–603, 11–13	EP713 transmitted to EU-5, EU-6, EU-60, EU-1 (three replicas for each pair tested)
Recipient.EP713	All of the EP713 recipients’ viral populations combined
CM	CR23 parental isolate in the vertical transmission (mother.CR23)
CS1-CS4	CR23 spores’ viral populations (one to four)
Spore.CR23	All of the CR23 spores’ viral populations combined
EM	Euro7 parental isolate in the vertical transmission (mother.Euro7)
ES1-ES5	Euro7 spores’ viral populations (one to five)
Spore.Euro7	All of the Euro7 spores’ viral populations combined
K1M	ks_30.4 parental isolate in the vertical transmission (mother.ks_30.4)
K1S1-K1S5	ks_30.4 spores’ viral populations (one to five)
Spore.ks_30.4	All of the ks_30.4 spores’ viral populations combined
K2M	ks_46.4 parental isolate in the vertical transmission (mother.ks_46.4)
K2S1-K2S5	ks_46.4 spores’ viral populations (one to five)
Spore.ks_46.4	All of the ks_46.4 spores’ viral populations combined
OM	oz_08.2 parental isolate in the vertical transmission (mother.oz_08.2)
OS1-OS6	oz_08.2 spores’ viral populations (one to six)
Spore.oz_08.2	All of the oz_08.2 spores’ viral populations combined
CP	Pooled.CR23 sample (CR23 mycelia not part of any transmission experiments representing a ‘baseline’ viral diversity of that sample
CD1, −2, −44, −5	CR23 donor isolate (EU-1) paired with EU-1, HK22C (EU-2), −44, −5 tester respectively
Donor.CR23	All of the CR23 donors’ viral populations combined
C11-C13, 21–23, 441–443, 51–53	CR23 transmitted to EU-1, HK22C (EU-2), −44, −5 tester (three replicas for each pair tested)
Recipient.CR23	All of CR23 recipients’ viral populations combined
EP	Pooled.Euro7 sample (Euro7 mycelia that was not part of the transmissions, i.e. Euro7 that grew alone and represents a ‘baseline’ of the viral diversity in that sample)
ED9, −15, −36, −17	Euro7 donor isolate (EU-9) paired with EU-9, −15, −36, −17 tester, respectively
Donor.Euro7	All of the Euro7 donors’ viral populations combined
E91-E93, 151–153, 361–363, 171–173	Euro7 transmitted to EU-9, −15, −36, −17 (three replicas for each pair tested)
Recipient.Euro7	All of the Euro7 recipients’ viral populations combined

For a more detailed list of analysed viral populations, see Supplementary Table S1.

### Vertical transmission

#### Viral transmission success rates

The percentage of successful vertical transmission of the virus was over 75%, and it didn’t differ significantly between CHV1 subtypes. Isolates CR23, Euro7, EP713, and field isolate ks_46.4 had a 100% transmission rate, i.e. all analysed conidia were infected with CHV1, while the other two field isolates, oz_08.2 and ks_30.4, had 77% and 92% CHV1 transmission rate into conidia, respectively.

#### Viral populations’ diversity changes

For all virus strains, there was no significant difference in the diversity between parental and progeny populations except in the case of ks_46.4 vertical transfer experiment. Pairwise comparison between analysed populations, both parental and progeny, revealed significant differences only between progeny populations ks.46.4_03 and ks.46.4_04 (*P*-value 0.019303), as well as ks.46.4_04 and ks46.4_10 (*P*-value 0.020605), which differed significantly from each other, but even these three populations did not differ significantly from their parental population.

In order to see if the vertical transmission had any effect on either the number of mutations or the number of viral variants, i.e. is the bottleneck effect visible in their reduction, the average numbers of mutations, i.e. the average number of all of the mutations (SNPs) observed at a certain genome position in a viral population and the average number of denoised viral variants (unique haplotypes) were observed separately for the parental viral populations and for the conidial progeny viral populations in the vertical transmission experiments (Table 2). There was a decline in both average number of mutations and number of viral variants between parental and conidial progeny populations of the subtype I, but surprisingly, while there was a sharp decline in the average number of mutations between parental and conidial progeny populations for the subtype F1, the number of viral variants actually slightly increased.

**Table 2:** The average number of mutations, i.e. the average number of all of the mutations (SNPs) observed at a certain genome position in a viral population and the average number of denoised viral variants in populations, before (parental viral populations) and after (conidial progeny viral populations) vertical transmission of CHV1 subtype I and subtype F1.

	Subtype I ^a^ Subtype F1^a^			
	Parental	Conidial progeny	Parental	Conidial progeny
Average number of mutations	20 ± 7.3	18.8 ± 9.9	270	12.2 ± 1.1
Average number of viral variants	39.8 ± 25.9	26.1 ± 26.1	211	229.8 ± 36.1

a There are five subtype I strains (Euro7, CR23, ks_30.4, ks_46.4, and oz_08.2) and one subtype F1 strain (EP713) included in this analysis.

**Figure 1:**
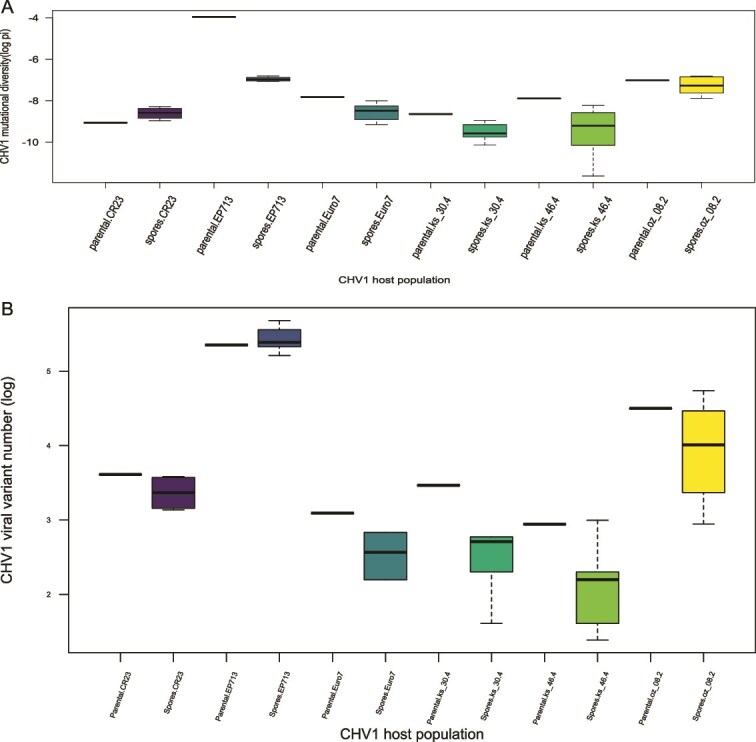
Change in mutational diversity (π) (a) and in the number of viral variants (b) after the vertical transmission. Mutational diversity and number of viral variants are log-transformed and given on the *y*-axis, while the CHV1 host population considered is on the *x*-axis. In this box and whisker plot, the box gives the interquartile range (IQR) and the median (the line in the box), while the whisker length is the default value of 1.5 × IQR. Values beyond these are plotted as outliers.

A bottleneck effect on the viral population was observed when CHV1 is transmitted into spores which is visible in the reduction of the overall mutational diversity (π): for the subtype F1 viral population (EP713), the π was 0.019105 in the parental viral population and the mean π in the conidial progeny dropped to 0.000955; for subtype I viral populations (Euro7, ks_30.4, ks_46.4, and oz_08.2), the π of the parental viral populations were 0.000400, 0.000176, 0.000376, and 0.000901, respectively, while the mean π of the conidial progeny replicates were 0.000208, 0.000081, 0.000120, and 0.000750. The reduction of π was visible in all populations except for CR23, where the π of the parental viral population (0.000116) slightly increased in its conidial progeny (mean π = 0.000189) (Fig. 1). However, this effect was significant only in the case of mutational diversity (π) reduction between EP713 parental isolate and its conidial progeny (*P*-value <0.001), but not for Nei’s H. There were no statistically significant differences between mutational diversity (π) or Nei’s H in any case for any of the I subtype virus strains tested. Bottleneck effect is also visible in the reduction of the number of viral variants transmitted to conidial progeny for all subtype I samples (Euro7, CR23, ks_30.4, ks_46.4, and oz_08.2) where the mean number of viral variants dropped from ~40 in the parental viral populations to ~26 in the conidial progeny (Table 2). Interestingly, in the subtype F1 (EP713), the mean number of viral variants increased in the conidial progeny from 211 to 230, despite the obvious drop in mutational diversity (π) (Fig. 1).

### Horizontal transmission

#### Viral transmission success rates

In the horizontal transmission experiment, there was a clear difference in the success rate of the transmission between fungal isolates heteroallelic at particular *vic* loci. As expected, heteroallelism at the *vic2* locus had the most restrictive effect, at *vic3* an intermediate effect, and at *vic4* there was essentially no effect on the CHV1 transmission rates. In all of the pairings with the vc tester isolates that have the same EU type as the donor, the transmission of the virus was 100% successful (Table 3).

**Table 3:** The percentage of viral transmission from the donor populations CR23, Euro7 and EP713 to the recipients heteroallelic on a particular *vic* locus. The EU vc types of the isolates are indicated in parentheses and recipient abbreviations explained in Supplementary Table S1.

	Donor viral populations (EU type) Donor abbreviations					
	CR23 (EU-1) CD1, CD2, CD44, CD5		Euro7 (EU-9) ED9, ED15, ED36, ED17		EP713 (EU-5) FD5, FD6, FD60, FD1	
Heteroallelic *vic* locus	Recipient EU type	Transmission efficiency (recipient abbreviation)	Recipient EU type	Transmission efficiency (recipient abbreviation)	Recipient EU type	Transmission efficiency (recipient abbreviation)
NONE	EU-1	100% (C11, C12, C13)	EU-9	100% (E91, E92, E93)	EU-5	100% (F51, F52, F53)
*vic2*	EU-2^a^	5.9% (C21, C22, C23)	EU-15	5.6% (E151, E152, E153)	EU-6	6.1% (F61, F62, F63)
*vic3*	EU-44	25% (C441, C442, C443)	EU-36	14.3% (E361, E362, E363)	EU-60	12.5% (F601, F602, F603)
*vic4*	EU-5	97.8% (C51, C52, C53)	EU-17	96% (E171, E172, E173)	EU-1	98.6% (F11, F12, F13)

a Isolate HK22C (EU-2) was used instead of standard vc-type tester.

The rate of horizontal transfer of CHV1 was not affected by the viral strain, as was determined by Fisher’s exact test (Supplementary Table S2). Overall, there was a slight but significant difference for CR23 (*P* =0.00157) in the CHV1 transfer rate between pairing of the isolates heteroallelic at *vic2* and those heteroallelic at *vic3*, although when including transfer rates of EP713 and Euro7 viral strains, the effect vanished (Supplementary Table S2).

#### Viral populations’ diversity changes

In the horizontal transmission experiment, the GLM test suggested no significant differences between any of the tested populations, i.e. none of the *vic* loci tested led to a change in viral population diversity.

The average number of mutations and the average number of denoised viral variants were calculated separately for the donors and the recipient viral populations in the horizontal transmission experiment but did not show any significant difference (Table 4.).

**Table 4:** The average number of mutations, i.e. the average number of all of the mutations (SNPs) observed at a certain genome position in a viral population and the average number of denoised viral variants in populations, before (donor viral populations) and after (recipient viral populations) horizontal transmission of CHV1 subtype I and subtype F1.

	Subtype I Subtype F1			
	Donor	Recipient	Donor	Recipient
Average number of mutations	28.5 ± 4	46.2 ± 70	12 ± 0.7	103.2 ± 127.6
Average number of viral variants	28 ± 5.8	25.3 ± 15	74.3 ± 21.4	73.7 ± 48.9

**Figure 2:**
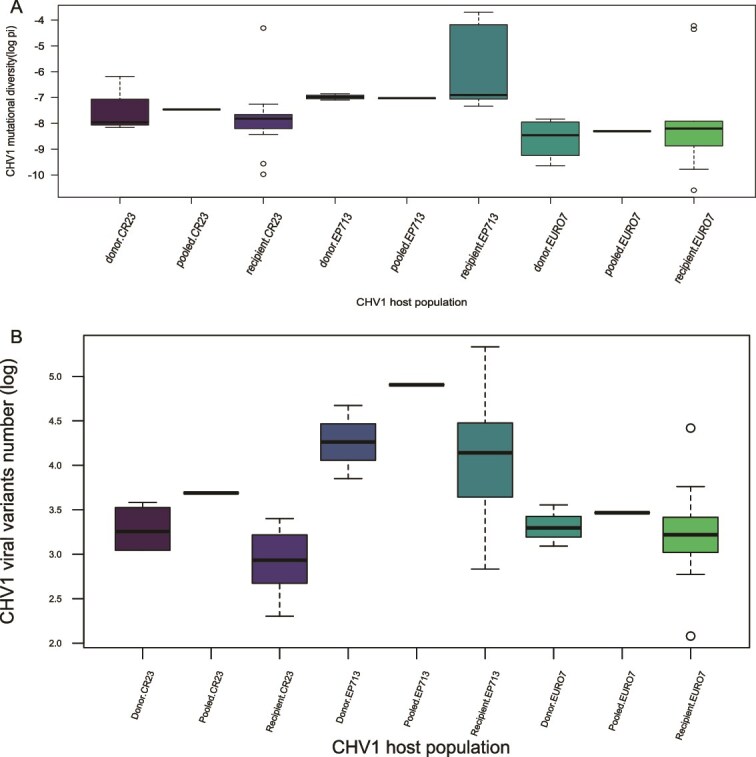
Change in mutational diversity (π) (a) and in the viral variant number (b) after the horizontal transmission. Mutational diversity and number of viral variants are log-transformed and given on the *y*-axis, while the CHV1 host population considered is on the *x*-axis. In this box and whisker plot, the box gives the interquartile range (IQR) and the median (the line in the box), while the whisker length is the default value of 1.5 × IQR. Values beyond these are plotted as outliers.

Interestingly, during the horizontal transmission of the viral populations, the bottleneck effect, i.e. drop in mutational diversity or number of detected viral variants, was not statistically significant for any of the viral strains tested, i.e. CR23, Euro7, and EP713, analysed in any of the pairings considered (Fig. 2.). The change in mutational diversity (π) or Nei’s H between donor populations and recipient populations was not significant, except for Nei’s H when the *vic3* locus was involved in horizontal transmission of the CHV1 for I subtype (*P*-value 0.01668) and for mutational diversity (π) when the *vic2* locus was considered for F subtype (*P*-value 0.03978).

### Inter-host population structure

**Figure 3:**
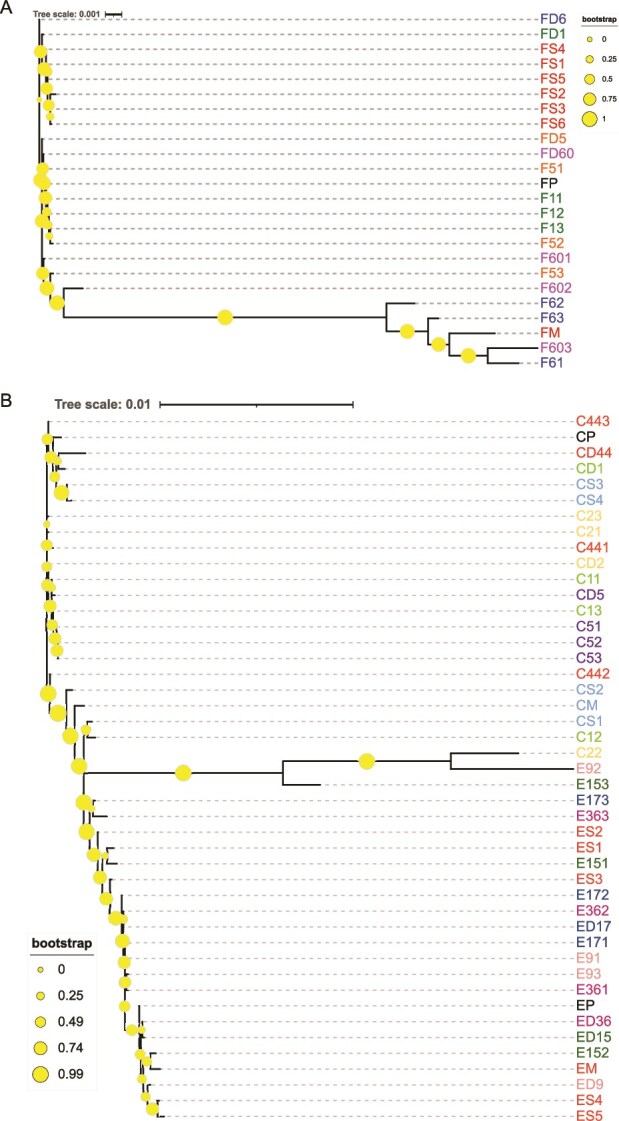
The consensus derived inter-host relationships as inferred by a RAxML-NG: (a) vertical and horizontal transmission of EP713 (subtype F1), (b) vertical and horizontal transmission of the Euro7 and CR23 (subtype I). Viral populations engaged in vertical transfer are in red (FM, CM, and EM are parental populations; FS1, FS2, FS3, FS4, and FS5, CS1, CS2, CS3, CS4, CS5, and CS6, and CS1, CS2, CS3, CS4, CS5, and CS6 are progenitor populations), viral populations engaged in horizontal transfer are given in various colour, with the same colour coding one particular set of experiments (i.e. testing for a particular *vic* locus heteroallelism). Prototypical viral populations not engaged in any experiment (i.e. controls) are in black. The phylogenetic tree is unrooted, and the bootstrap values are given as circles on the nodes. The abbreviations of the viral populations are given in Supplementary Table S1.

For the phylogenetic analysis of the inter-host population structure, made with RAxML-NG, the CHV1 host populations were grouped based on the different types of transmission—vertical or horizontal, and on the different CHV1 subtype—subtype I (CR23 and Euro7) and subtype F1 (EP713). The results are given as phylogenetic trees and show that in most cases, after either vertical or horizontal transfer, the particular set of populations derived from a certain prototypical viral strain group together, i.e. Euro7- and CR23-derived viral populations are usually more closely related to each other than to viral populations derived from the other strains (Fig. 3). The only exceptions were F61, F62, and F63 viral populations, which did not group with their donor FD6 viral population (Fig. 3a) and C22 recipient viral population that grouped with Euro7 derived viral populations, rather than its donor CD2 (Fig. 3b). In both cases, the horizontal transfer of the viral populations was performed between isolates heteroallelic at the *vic2* locus.

Generally, in vertical transmission, EP713 conidial progeny populations tend to group closely together (Fig. 3a), something that is not really observed for Euro7 or CR23 (Fig. 3b). In the horizontal transmission experiment grouping of the CHV1 recipient viral populations from the same sets of experiments is more evident for the EP713 viral strain (Fig. 3a), but again, it is not as pronounced for subtype I viral strains Euro7 and CR23 (Fig. 3b).

For the vertical transmission of subtype F1 (EP713) in all our analysed viral populations, i.e. one parental and six conidial progeny populations, we observed 1150 different viral variants, but only 19 were shared among the parental population and their offspring. When analysing viral populations derived from the CR23 strain, 91 different viral variants were identified, and five of them are present in all populations. Two variants comprise >65% of the total number of viral variants. In viral populations derived from the Euro7 prototypical strain, 51 different viral variants were present, and two variants are present in almost all analysed viral populations where they comprise >90% of the total number of viral variants, except in one: ES3, where they comprise >79%.

For the horizontal transmission of subtype F1 (EP713), an average of four variants is represented with at least 5% of the total number of viral variants per population. In the horizontal transmission experiments of the CR23 viral strain, only one variant was present in all analysed viral populations, while in the horizontal transmission of Euro7, there was not a single variant that appeared in all of our analysed viral populations. The number of different variants, along with the number of the most common ones, is given in Table 5.

**Table 5:** Total number of different variants identified for each strain in analysed viral populations in the vertical and horizontal transmission experiments; mean number of variants in analysed viral populations is given in brackets. The average number of the most common viral variants represented with at least 5% of the total number of viral variants is also presented.

	EP713		CR23		EURO7		KS_30.4	KS_46.4	OZ_08.2
	Vertical	Horizontal	Vertical	Horizontal	Vertical	Horizontal	Vertical	Vertical	Vertical
Number of different variants (Mean number of variants)	1 150 (227.1)	811 (77.4)	91 (30.8)	222 (22.8)	51 (19.7)	309 (30.4)	63 (15.7)	46 (11.2)	362 (64.3)
Number of variants represented in at least 5% of the total number	5	4	3	2.8	2.5	3.3	1.7	2.85	2.7
Number of analysed populations	7	17	5	17	6	17	6	6	7

### Intra-host patterns of selection

The signals of selection were characterized using synonymous and nonsynonymous (missense) mutations, more specifically, mutational diversity (π) was compared between synonymous (hereafter πS) and nonsynonymous (hereafter πN) mutations in our 5 kb amplicon. When considering all analysed viral populations, πS was greater than πN in 79 out of 88 analysed viral populations (πS = 0.002321 ± 0.006485, πN = 0.000356 ± 0.000654, paired *t*-test, *t* = −3.1328, mean difference = 0.001965, *P*-value  0.002359), while πN > πS is rare and appears in the other 9 viral populations (Supplementary Table S3). When considering the two analysed ORFs separately, in ORFA 60 and ORFB 58, out of the total of 88 samples had πS > πN.

In vertical transmission experiments, πS was greater than πN in 29 out of 37 analysed viral populations but the difference was not significant (πS = 0.000905 ± 0.003988, πN = 0.000224 ± 0.00045, paired *t*-test, *t* = −1.1458, mean difference = 0.000681, *P*-value 0.25944). Only 1 (OZM) out of 9 parental or prototypical populations had πN > πS, while 7 out of 31 conidial progeny viral populations had πN > πS.

For strains from the horizontal transmission experiment, πS was greater than πN in 50 out of 51 analysed viral populations and this was statistically more significant (πS = 0.003348 ± 0.00765, πN = 0.000451 ± 0.000755, paired *t*-test, *t* = −2.9634, mean difference = 0.002897, *P*-value  0.004649). In the horizontal transmission assays, none of the donor populations had πN > πS and only one (E173) recipient population had πN > πS.

Missense mutations, in which the codon changes, are presumed to have a moderate impact, i.e. can change the protein effectiveness, were found in all of our samples. Frameshift mutations that are presumed to have a highly disruptive impact on the protein, probably causing protein truncation and loss of function or triggering nonsense-mediated decay were also found in some samples. Deletions were also detected that are likely to represent defective viral RNAs. Frameshift mutations were detected on five different genomic positions in the EP713 parental viral population, as well as in all three replicates of EP713 recipient populations that differ at *vic2* and one that differs at the *vic3* locus*.* An additional frameshift mutation was present in two recipient populations: F61 and F603. Nine frameshift mutations have been observed in CR23 and Euro7 viral strain populations across all the experiments. At genomic position 4672 (ORFB), a conservative 237 bp long deletion is present in almost all subtype F1 viral populations including the parental and donor populations (21/24) where it appears at high, ~65%, intra-host frequency. The same deletion was observed at position 4671 (ORFB) in one replicate isolate of the subtype I (Euro7—E153), albeit at a low intra-host frequency of 13,3%. A very large conservative 978 bp long deletion at genomic position 2389 (ORFB) is present in all Euro7 viral populations including both parental and conidial progeny viral populations analysed in the vertical transmission experiments with an average frequency of ~65%, and in one Euro7 donor population (ED15) in the horizontal transmission experiment at 13% frequency. Another large 915 bp long conservative deletion was also present at genomic position 2409 (ORFB) in one replicate in the vertical transmission, K2S1, at 45.1% frequency.

In the horizontal transmission experiment, we also noticed differences in the transmission efficiency of certain viral variants, considering different *vic* loci heteroallelism. When all of the alleles at the *vic* loci were the same or the only difference was in *vic4*, the number of transmitted variants was relatively high: for EP713 39% and 45%, for CR23 31% and 30%, and for Euro7 38% and 53%, respectively. In comparison, in donor/recipient pairs that were heteroallelic at either *vic2* or *vic3*, the number of transmitted variants was lower: for EP713 13% and 20%, for CR23 29% and 19%, and for Euro7 23% and 17%, respectively.

## Discussion

In order to be sustained in nature, the virus CHV1 has to be efficiently transmitted to an uninfected fungus. The results of this study are in concordance with those of Peever et al. (2000), which showed that the rate of vertical transmission of CHV1 was >95% and that there was no statistical difference in the rate of CHV1 transmission into the conidia between different viral strains. Many other mycoviruses are efficiently transmitted vertically into the spores, for example, Wang et al. (2016) reported a 100% transmission rate into ascospores of Fusarium graminearum hypovirus 1, though the transmission rates into conidia and ascospores of several other dsRNA mycoviruses isolated from *Fusarium graminearum* were between 30% and 100% (Chu et al. 2004). In basidiomycetes, high vertical transmission rates of viruses were observed as well, for example, Lentinula edodes mycovirus was transmitted in 9 out of 10 basidiospores (Won et al. 2013). It is worth noting that CHV1 is transmitted only into asexual spores, i.e. conidia, but the host, *C. parasitica,* has been observed to reproduce sexually as well (Dutech et al. 2010), which can hinder CHV1 transmission in nature.

Successful horizontal transmission of CHV1 between hypovirus-infected and virulent *C. parasitica* isolates is around 100% when the vc type of the isolates is the same (Cortesi and Milgroom 1998, Cornejo et al. 2019), while that number is significantly lower (0%–50%) between different vc types, especially when heteroallelism at the *vic2* locus is involved (Liu and Milgroom 1996). Cortesi et al. (2001) showed that CHV1 was successfully transmitted in 21% of the pairings that were heteroallelic at *vic2*, 76% when they were heteroallelic at *vic3*, while no restriction of the virus transmission was observed when heteroallelic at *vic4* or when they had the same *vic* genotype. Our results show the same general trend as the previous research with *vic2* showing the strongest inhibition, *vic3* a more intermediate one, and *vic4* weak or no inhibition of CHV1 transfer, thus confirming the restrictive effect of heteroallelism at particular *vic* loci on virus transmission, observed previously (Cortesi et al. 2001, Papazova-Anakieva et al. 2008).

The first quantitative estimate of a population bottleneck during virus transmission was obtained for an insect-borne plant virus, the Potato virus Y (PVY), transmitted by aphids. A stochastic model was then used to estimate that each aphid could transmit 0.5–3.2 viral genomes on average (Moury et al. 2007). Population bottlenecks during contact transmission of tomato mosaic virus (ToMV) have also been analysed, and the number of viral genomes initiating infection has been estimated between 1 and 4 (Sacristán et al. 2011). In Leigh et al. (2021), the mean number of CHV1 variants that infect a chestnut blight canker and initiate a new intra-host population was 1.99 ± 1.51. Most of the viral variants from a canker were highly related, indicating significant bottleneck events during the virus transmission and expansion of the viral population afterwards. Similarly, in our experiments, a reduction of the viral diversity was observed in vertical virus transmission. The mutational diversity (π) and the number of viral variants were reduced in the conidial progeny viral populations when compared to their parental populations. Furthermore, the resulting viral variants were highly similar among themselves, more than to the parental population from which they diverged.

In the study on Cucumber mosaic virus (CMV), the horizontal transmission *via* aphids was studied (Ali et al. 2006). The authors found that the bottleneck occurred during the inoculation period and not during the acquisition period of the virus. Similarly, we observed a bottleneck when the viral populations were transmitted into spores, indicated by a decrease in the number of mutations and the overall number of viral variants. However, the reduction of mutational diversity and number of viral variants was not as pronounced in the horizontal transfer of the virus, suggesting that the most pronounced drop in viral population diversity occurs during the vertical transfer of the viral populations into the spores, rather than in the horizontal transfer of the virus between two fungal individuals. In nature, the hypovirus first spreads vertically when a viral inoculum is delivered into an active canker *via* spores, and only later spreads horizontally inside a canker, while the artificially initiated biocontrol is based on the horizontal transmission, i.e. the virus is transmitted by inoculating a large amount of hypovirus-infected mycelia into a chestnut blight canker, which then delivers the viral inoculum. In combination with the finding of Leigh et al. (2021) that the new viral populations in cankers are usually initiated by a small number of viruses, our results support the former, i.e. spread *via* conidia, as the more prominent way of natural dissemination of CHV1.

To determine the nature of viral evolution following systemic movement through a plant, Dunham et al. 2014 sequenced 24 leaves of *Cucurbita pepo* vine that was infected with zucchini yellow mosaic virus (ZYMV). Out of 112 genetic variants observed in the data set, which included viral population in the mechanically inoculated leaf and the viral populations that spread into 23 other leaves along the same vine, only a few were present in the plant postinfection; therefore, the authors concluded that systemic movement of the virus is characterized by sequential population bottlenecks. It is unclear whether this is due to selection, i.e. removing deleterious mutations, genetic drift, or a combination of both. One explanation for this finding is that most genotypes generated during plant colonization were deleterious and thus quickly lost. However, the authors suggested that the major drivers of ZYMV genetic diversity were neutral evolution and genetic drift because most observed mutations were synonymous. Similarly, our research, as πS > πN in most analysed viral populations, suggests that the intra-host CHV1 population diversity is primarily shaped by purifying selection. Viral populations that had πN > πS were less common, which can be a sign of positive selection (Nelson et al. 2015). Alternatively, it can also suggest load accumulation or simply be a result of the fact that selection did not have the time to act yet, since in our experiments, it is most commonly observed in viral populations derived from spores. These viral populations are relatively young, as they are believed to be initiated by only a few viral variants that expanded rapidly into a new viral population of a growing mycelium. Interestingly, even though purifying selection entails selective removal of harmful, i.e. deleterious, mutations, in our experiments, we found three large deletions, present at frequencies ranging from 13 to 80%, in viral populations from prototypic isolates Euro7 and EP713 and field isolate ks_30.4. Two of these three large deletions were found in both the parental and donor viral populations, as well as in some of the conidial progeny and recipient viral populations. A third large deletion was not present in the parental ks_30.4 field isolate but was found in one of its conidial progeny viral population: K2S1. Defective viral RNAs have previously been described in some lab strains of CHV1, and were therefore thought only to arise after the relaxation of selection and repeated bottleneck events endured during prolonged laboratory fungal culturing (Shapira et al. 1991) such as the case for prototypical isolates EP713 and Euro7. Viruses can generate modified forms of their genome during their replication as tools to adapt to environmental challenges. Molecular characterizations of CHV1 RNAs revealed the accumulation of significant levels of defective interfering (DI) RNAs (Tartaglia et al. 1986, Shapira et al. 1991). DI RNAs originate from the parental viral RNA genome as a result of recombination and/or deletion events. The presence of DI RNAs results in the suppression of parental RNA accumulation, leading to attenuation of symptoms (Simon et al. 2004) and persistent virus infections (Huang and Baltimore 1970). Such a deletion in a field isolate of CHV1 could, in concordance with previous research, reduce the virulence of CHV1 towards its fungal host to ensure that the virus gets transmitted and to promote its persistence in a host.

For animal viruses, studies showed that a single HIV variant is usually the origin of a viral population within a patient (Gijsbers et al. 2012). Similarly, one or two founder viral variants constituted the founder population in four individuals with Hepatitis C virus infection (Bull et al. 2011). This is consistent with previous research on CHV1 where the mean number of infecting founder viruses was estimated to be 1.99 ± 1.51 (Leigh et al. 2021). In our vertical transmission experiment, we observed that slightly fewer viral variants were transmitted from the parental viral population to the conidial progeny viral populations in the field samples (12%–20%) in comparison to the prototypic viral strains (24%–33%). In the horizontal transmission experiment, we also noticed that there was a difference in the percentage of shared viral variants between donor and recipient CHV1 populations when we considered the different *vic* loci. For all our samples, when the alleles on all *vic* loci were the same or when fungal isolates were heteroallelic at *vic4*, the number of shared viral variants ranged from 30% to 53%. In comparison, when the heteroallelic locus was either *vic2* or *vic3*, the proportion of shared viral variants dropped: for EP713, 13 and 20%, for Euro7, 23 and 17%, and for CR23, 29 and 19%, respectively, which further corroborates the effect of these *vic* loci on viral transmission (Cortesi et al. 2001). The differences in viral transmission are correlated with allele-specific differences in the rate of programmed cell death (PCD) associated with incompatible interactions, in which delayed PCD allows greater virus transmission (Biella et al. 2002).

Narrow bottlenecks might not always happen during virus transmission. For example, in the case of the Dengue virus, Aaskov et al. (2006) showed that defective viral genotypes are transmitted among humans and mosquitoes in nature. The suggested explanation was that the defective genotypes are complemented by functional ones and that relaxed transmission bottlenecks allow the transmission of both types in the recipient host. The absence of a severe transmission-associated bottleneck was observed in a study analysing changes in population diversity of the Equine influenza virus between donor and recipient horses as well (Murcia et al. 2010). The reason for that was suggested to be a large number of transmission events that were able to transmit a large viral population, despite, presumably, a significant bottleneck effect associated with a single transmission event. Therefore, whether the transmission bottleneck will be severe or relaxed cannot be exclusively linked to a specific mode of natural viral transmission (Gutiérrez *et al*., 2012). Since we observed a more pronounced bottleneck effect in the vertical transmission experiment, than in the horizontal transmission experiment, we conclude that the number of viral variants that can be transmitted may be limited during the formation of the spores, as opposed to the hyphal anastomosis formation where the hyphae of the recipient are in prolonged contact with the mycelia of the donor, thus allowing transmission of more viral variants, especially when there is no physiological or genetic barrier to prevent formation of stable anastomosis. Interestingly, while a reduction of mutational diversity and the number of viral variants were detected in vertical transfer experiments, the resulting viral population compositions were not significantly different from their respective parental population, indicating preservation of the most viral variants during the transmission.

In the study of SARS-CoV-19 (Lythgoe et al. 2021), only one or two viral variants were observed in most samples, while only a few of the samples carried many variants. Even though the results indicate strong purifying selection, the authors also saw evidence for transmission clusters associated with households and other possible superspreader events. After being transmitted, most viral variants were lost, but occasionally, some were involved in ongoing transmission and dissemination. In contrast, influenza A is characterized by a high genetic diversity. In research done by Poon et al. (2016), the same variants were found in multiple members of the community. While the relative frequencies of variants fluctuated, patterns of genetic variation were more similar within than between households. In most of our analysed viral populations, we have a few dominant viral variants that appear in the majority of intra and inter-host viral populations. Unsurprisingly, due to the role of random chance in transmission bottlenecks, these dominant viral variants are the ones that are most frequently transmitted, while most of the variants present in an analysed viral population are very rare.

## Conclusion

In our research, the intra-host population diversity of CHV1 before and after vertical and horizontal transmission was assessed using state-of-the-art NGS technology. Two diversity metrics were used primarily to describe the viral diversity within a host: mutational diversity (π) and the viral variants diversity (Nei’H). A pronounced bottleneck for most of our populations was observed after the vertical but not after horizontal transmission, visible in the drop in mutational diversity and the number of viral variants. This strongly suggests that the number of viral variants that can be transmitted is limited during the formation of the spores, as opposed to the hyphal anastomosis, which allows prolonged time of contact between different mycelia and subsequently allows the transmission of more viral variants. In concordance with previous findings on the frequency of hypovirus transmission, vegetative incompatibility also affected intra-host diversity during horizontal virus transmission. A smaller number of viral variants were transmitted if donor and recipient populations were heteroallelic at the *vic2* and *vic3* loci compared to when isolates had the same *vic* genotype or were heteroallelic at the *vic*4 loci. In contrast to prototypic strains that might have accumulated more mutations over the years because of repeated subculturing and freeze–thaw cycles, viral populations from the two newly sampled field isolates from Kašt, as well as their conidial progeny populations, had the lowest nucleotide and viral variant diversity. Finally, we hypothesize that the intra-host CHV1 population diversity is primarily shaped by purifying selection, i.e. πS > πN, although we found evidence of severe mutations, such as frameshift mutations and deletions, with potentially high biological impact. Major deletions in genomes of field isolates could reduce virulence of CHV1 toward its fungal host but increase its chances of being transmitted. Finally, our work showed that most of the variants present in an analysed viral population are very rare, while a few dominant variants are the ones that are most commonly transmitted.

## Supplementary Material

suppl_Table_veaf082

## Data Availability

The original data are openly available at https://www.ncbi.nlm.nih.gov/sra/PRJNA982774.
