# Supplementary material for: Changes in intra-host mycovirus population diversity after vertical and horizontal transmission
Source: Virus Evol. 2025 Oct 23;11(1):veaf082. doi: 10.1093/ve/veaf082 (PMC12611243; doi:10.1093/ve/veaf082)
Supplement: suppl_Table_veaf082 [file suppl_table_veaf082.zip › suppl_Table_veaf082/suppl_Table S1.docx]

Table S1. The list of all viral analysed populations and abbreviations used throughout the paper.

| Viral strain | Abbreviation | Parental (vertical transfer) or donor (horizontal transfer) isolates’ name | Progeny (vertical transfer) or recipient (horizontal transfer) isolates’ name |
| --- | --- | --- | --- |
| EP713 | FP | Pooled.EP713 sample i.e., EP713 mycelia not part of any transmission experiments representing a "baseline" viral diversity of that sample | - |
|  | VERTICAL TRANSFER | | |
|  | FM | Mother.EP713 sample i.e., EP713 parental isolate in the vertical transmission experiments | - |
|  | FS1 | - | EP713 first spore viral population |
|  | FS2 | - | EP713 second spore viral population |
|  | FS3 | - | EP713 third spore viral population |
|  | FS4 | - | EP713 fourth spore viral population |
|  | FS5 | - | EP713 fifth spore viral population |
|  | FS6 | - | EP713 sixth spore viral population |
|  | Spore.EP713 | - | All of the EP713 spores’ viral populations listed above combined |
|  | HORIZONTAL TRANSFER | | |
|  | FD5 | EP713 donor isolate paired with EU-5 tester (*same EU-type*) | - |
|  | F51 |  | EP713 transmitted to EU-5 - first replica |
|  | F52 |  | EP713 transmitted to EU-5 - second replica |
|  | F53 |  | EP713 transmitted to EU-5 - third replica |
|  | FD6 | EP713 donor isolate paired with EU-6 tester (*vic2* difference) | - |
|  | F61 | - | EP713 transmitted to EU-6 - first replica |
|  | F62 | - | EP713 transmitted to EU-6 - second replica |
|  | F63 | - | EP713 transmitted to EU-6 - third replica |
|  | FD60 | EP713 donor isolate paired with EU-60 tester (*vic3* difference) | - |
|  | F601 | - | EP713 transmitted to EU-60 - first replica |
|  | F602 | - | EP713 transmitted to EU-60 - second replica |
|  | F603 | - | EP713 transmitted to EU-60 - third replica |
|  | FD1 | EP713 donor isolate paired with EU-1 tester (*vic4* difference) | - |
|  | F11 | - | EP713 transmitted to EU-1 - first replica |
|  | F12 | - | EP713 transmitted to EU-1 - second replica |
|  | F13 | - | EP713 transmitted to EU-1 - third replica |
|  | Donor.EP713 | All of the EP713 donors’ viral populations listed above combined | - |
|  | Recipient.EP713 |  | All of the EP713 recipients’ viral populations listed above combined |
| CR23 | CP | Pooled.CR23 sample (CR23 mycelia not part of the transmissions, i.e. CR23 that grew alone and represents a "baseline" of the viral diversity in that sample) | - |
|  | VERTICAL TRANSFER | | |
|  | CM | CR23 parental isolate in the vertical transmission (mother.CR23) | - |
|  | CS1 | - | CR23 first spore |
|  | CS2 | - | CR23 second spore |
|  | CS3 | - | CR23 third spore |
|  | CS4 | - | CR23 fourth spore |
|  | Spore.CR23 | - | All of the CR23 spores (listed above) |
|  | HORIZONTAL TRANSFER | | |
|  | CD1 | CR23 donor isolate paired with EU-1 tester (*same EU-type*) | - |
|  | C11 | - | CR23 transmitted to EU-1 - first replica |
|  | C12 | - | CR23 transmitted to EU-1 - second replica |
|  | C13 | - | CR23 transmitted to EU-1 - third replica |
|  | CD2 | CR23 donor isolate paired with HK22C (*vic2* difference) | - |
|  | C21 | - | CR23 transmitted to HK22C - first replica |
|  | C22 | - | CR23 transmitted to HK22C - second replica |
|  | C23 | - | CR23 transmitted to HK22C - third replica |
|  | CD44 | CR23 donor isolate paired with EU-44 tester (*vic3* difference) | - |
|  | C441 | - | CR23 transmitted to EU-44 - first replica |
|  | C442 | - | CR23 transmitted to EU-44 - second replica |
|  | C443 | - | CR23 transmitted to EU-44 - third replica |
|  | CD5 | CR23 donor isolate paired with EU-5 tester (*vic4* difference) | - |
|  | C51 | - | CR23 transmitted to EU-5 - first replica |
|  | C52 | - | CR23 transmitted to EU-5 - second replica |
|  | C53 | - | CR23 transmitted to EU-5 - third replica |
|  | Donor.CR23 | All of the CR23 donors’ viral populations listed above combined | - |
|  | Recipient.CR23 | - | All of the CR23 recipients’ viral populations listed above combined |
| Euro7 | EP | Pooled.Euro7 sample (Euro7 mycelia not part of the transmissions, i.e. Euro7 that grew alone and represents a "baseline" of the viral diversity in that sample) | - |
|  | VERTICAL TRANSFER | | |
|  | EM | Euro7 parental isolate in the vertical transmission (mother.Euro7) | - |
|  | ES1 | - | Euro7 first spore |
|  | ES2 | - | Euro7 second spore |
|  | ES3 | - | Euro7 third spore |
|  | ES4 | - | Euro7 fourth spore |
|  | ES5 | - | Euro7 fifth spore |
|  | Spore.Euro7 | - | All of the Euro7 spores (listed above) |
|  | HORIZONTAL TRANSFER | | |
|  | ED9 | Euro7 donor isolate paired with EU-9 tester (*same EU-type*) | - |
|  | E91 | - | Euro7 transmitted to EU-9 - first replica |
|  | E92 | - | Euro7 transmitted to EU-9 - second replica |
|  | E93 | - | Euro7 transmitted to EU-9 - third replica |
|  | ED15 | Euro7 donor isolate paired with EU-15 tester (*vic2* difference) | - |
|  | E151 | - | Euro7 transmitted to EU-15 - first replica |
|  | E152 | - | Euro7 transmitted to EU-15 - second replica |
|  | E153 | - | Euro7 transmitted to EU-15 - third replica |
|  | ED36 | Euro7 donor isolate paired with EU-36 tester (*vic3* difference) | - |
|  | E361 | - | Euro7 transmitted to EU-36 - first replica |
|  | E362 | - | Euro7 transmitted to EU-36 - second replica |
|  | E363 | - | Euro7 transmitted to EU-36 - third replica |
|  | ED17 | Euro7 donor isolate paired with EU-17 tester (*vic4* difference) | - |
|  | E171 | - | Euro7 transmitted to EU-17 - first replica |
|  | E172 | - | Euro7 transmitted to EU-17 - second replica |
|  | E173 | - | Euro7 transmitted to EU-17 - third replica |
|  | Donor.Euro7 | All of the Euro7 donors (listed above) | - |
|  | Recipient.Euro7 | - | All of the Euro7 recipients (listed above) |
|  | VERTICAL TRANSFER | | |
| ks_30.4 | K1M | ks_30.4 parental isolate in the vertical transmission (mother.ks_30.4) | - |
|  | K1S1 | - | ks_30.4 first spore |
|  | K1S2 | - | ks_30.4 second spore |
|  | K1S3 | - | ks_30.4 third spore |
|  | K1S4 | - | ks_30.4 fourth spore |
|  | K1S5 | - | ks_30.4 fifth spore |
|  | Spore. ks_30.4 | - | All of the ks_30.4 spores (listed above) |
|  | VERTICAL TRANSFER | | |
| ks_46.4 | K2M | ks_46.4 parental isolate in the vertical transmission (mother.ks_46.4) | - |
|  | K2S1 | - | ks_46.4 first spore |
|  | K2S2 | - | ks_46.4 second spore |
|  | K2S3 | - | ks_46.4 third spore |
|  | K2S4 | - | ks_46.4 fourth spore |
|  | K2S5 | - | ks_46.4 fifth spore |
|  | Spore.ks_46.4 | - | All of the ks_46.4 spores (listed above) |
|  | VERTICAL TRANSFER | | |
| oz_08.2 | OM | oz_08.2 parental isolate in the vertical transmission (mother.oz_08.2) | - |
|  | OS1 | - | oz_08.2 first spore |
|  | OS2 | - | oz_08.2 second spore |
|  | OS3 | - | oz_08.2 third spore |
|  | OS4 | - | oz_08.2 fourth spore |
|  | OS5 | - | oz_08.2 fifth spore |
|  | OS6 | - | oz_08.2 sixth spore |
|  | Spore. oz_08.2 | - | All of the oz_08.2 spores (listed above) |
