# Supplementary material for: Changes in intra-host mycovirus population diversity after vertical and horizontal transmission
Source: Virus Evol. 2025 Oct 23;11(1):veaf082. doi: 10.1093/ve/veaf082 (PMC12611243; doi:10.1093/ve/veaf082)
Supplement: suppl_Table_veaf082 [file suppl_table_veaf082.zip › suppl_Table_veaf082/suppl_Table S2.docx]

Table S2. Fisher exact test, calculated to test if the difference between the viral populations in the recipients that were heteroallelic from the donor isolates at a particular *vic* locus compared to the ones that were heteroallelic at a different *vic*, has an effect on the success rate of the horizontal transmission.

|  |  | heteroallelic *vic* locus between donor and recipient | | | | Difference in CHV1 transfer rate between donor and recipient heteroallelic at different *vic* loci | | | | | |
| --- | --- | --- | --- | --- | --- | --- | --- | --- | --- | --- | --- |
| Hypovirulent donor isolate | recipient after pairing | *vic2* | *vic3* | *vic4* | none | *vic2*/*vic3* | *vic2*/*vic4* | *vic2*/none | *vic3*/*vic4* | *vic3*/none | *vic4*/none |
| CR23 | hypovirulent | 8 | 10 | 44 | 10 |  |  |  |  |  |  |
|  | virulent | 126 | 30 | 1 | 0 | 0,0015738 | 2,04x10^-32^ | 5,70x10^-11^ | 2,59x10^-13^ | 1,80x10^-05^ | 1 |
| Euro7 | hypovirulent | 7 | 10 | 24 | 10 |  |  |  |  |  |  |
|  | virulent | 119 | 60 | 1 | 0 | 6,06x10^-02^ | 1,35x10^-20^ | 4,58x10^-11^ | 1,45x10^-13^ | 1,12x10^-07^ | 1 |
| EP713 | hypovirulent | 10 | 20 | 71 | 10 |  |  |  |  |  |  |
|  | virulent | 154 | 140 | 1 | 0 | 5,56x10^-02^ | 4,67x10^-48^ | 3,43x10^-11^ | 6,41x10^-40^ | 7,08x10^-09^ | 1 |
| ALL | hypovirulent | 25 | 40 | 139 | 30 |  |  |  |  |  |  |
|  | virulent | 399 | 230 | 3 | 0 | 1,46x10^-04^ | 2,10x10^-101^ | 4,23x10^-32^ | 4,10x10^-68^ | 3,20x10^-22^ | 1 |
