# Supplementary material for: Changes in intra-host mycovirus population diversity after vertical and horizontal transmission
Source: Virus Evol. 2025 Oct 23;11(1):veaf082. doi: 10.1093/ve/veaf082 (PMC12611243; doi:10.1093/ve/veaf082)
Supplement: suppl_Table_veaf082 [file suppl_table_veaf082.zip › suppl_Table_veaf082/suppl_Table S3.docx]

Table S3. Wilcoxon test, calculated to see if there is a significant difference between viral populations after horizontal transmission, i.e. recipients based on the difference in the *vic* loci.

|  | same/*vic2* | same/*vic3* | same/*vic4* | *vic2*/*vic3* | *vic2*/*vic4* | *vic3*/*vic4* |
| --- | --- | --- | --- | --- | --- | --- |
| p (t test) | 0,10715 | 0,90754 | 0,33203 | 0,10209 | 0,015338* | 0,47696 |
| p (Wilcoxon test) | 0,20398 | 0,29974 | 0,12843 | 0,03945* | 0,02761* | 0,36118 |
