# Supplementary material for: Changes in intra-host mycovirus population diversity after vertical and horizontal transmission
Source: Virus Evol. 2025 Oct 23;11(1):veaf082. doi: 10.1093/ve/veaf082 (PMC12611243; doi:10.1093/ve/veaf082)
Supplement: suppl_Table_veaf082 [file suppl_table_veaf082.zip › suppl_Table_veaf082/suppl_Table S4.docx]

Table S4. Mutational diversity (π) compared between synonymous (πS) and non-synonymous (πN) mutations for all analysed viral populations

| sample | Donor (D)/parental (M) isolate, recipient (R)/spore (S) isolate or pooled isolate (P) | πN | πS | Larger π value |
| --- | --- | --- | --- | --- |
| CP | P | 0,000174 | 0,000456 | S |
| C11 | R | 0,000161 | 0,000178 | S |
| C12 | R | 0,000105 | 0,000336 | S |
| C13 | R | 0,00013 | 0,000407 | S |
| C51 | R | 0,000143 | 0,000294 | S |
| C52 | R | 0,0001 | 0,000289 | S |
| C53 | R | 0,000105 | 0,000306 | S |
| C441 | R | 0,000141 | 0,000795 | S |
| C442 | R | 7,17E-05 | 0,000156 | S |
| C443 | R | 2,33E-05 | 4,91E-05 | S |
| C21 | R | 1,42E-05 | 3,69E-05 | S |
| C22 | R | 0,00172 | 0,018248 | S |
| C23 | R | 0,000167 | 0,000306 | S |
| CD1 | D | 8,38E-05 | 0,000234 | S |
| CD5 | D | 0,000123 | 0,000229 | S |
| CD44 | D | 0,00049 | 0,002087 | S |
| CD2 | D | 7,43E-05 | 0,000372 | S |
| ED9 | D | 0,000101 | 0,000231 | S |
| ED15 | D | 3,43E-05 | 0,000142 | S |
| ED17 | D | 1,6E-05 | 6,34E-05 | S |
| ED36 | D | 0,000128 | 0,00029 | S |
| EP | P | 8,89E-05 | 0,000152 | S |
| E91 | R | 0,000123 | 0,000251 | S |
| E92 | R | 0,002014 | 0,019452 | S |
| E93 | R | 8,27E-05 | 9,53E-05 | S |
| E151 | R | 0,000112 | 0,000282 | S |
| E152 | R | 6,58E-05 | 0,000273 | S |
| E153 | R | 0,001442 | 0,018018 | S |
| E171 | R | 0,000106 | 0,000145 | S |
| E172 | R | 1,21E-05 | 6,04E-05 | S |
| E173 | R | 5,06E-05 | 1,72E-05 | N |
| E361 | R | 7,2E-05 | 0,00011 | S |
| E362 | R | 9,12E-05 | 0,000322 | S |
| E363 | R | 5,38E-06 | 2,71E-05 | S |
| FD1 | D | 0,000341 | 0,000365 | S |
| FD5 | D | 0,000312 | 0,00061 | S |
| FD6 | D | 0,000362 | 0,000503 | S |
| FD60 | D | 0,000381 | 0,000649 | S |
| FP | P | 0,000271 | 0,000703 | S |
| F11 | R | 0,000189 | 0,000547 | S |
| F12 | R | 0,000252 | 0,000582 | S |
| F13 | R | 0,00028 | 0,000578 | S |
| F51 | R | 0,000307 | 0,000611 | S |
| F52 | R | 0,00036 | 0,000478 | S |
| F53 | R | 0,000412 | 0,000611 | S |
| F601 | R | 0,000271 | 0,000682 | S |
| F602 | R | 0,000714 | 0,000863 | S |
| F603 | R | 0,003715 | 0,03172 | S |
| F61 | R | 0,002207 | 0,023801 | S |
| F62 | R | 0,002537 | 0,024735 | S |
| F63 | R | 0,001734 | 0,018019 | S |
| FM | M | 0,002747 | 0,024812 | S |
| FS1 | S | 0,00038 | 0,00044 | S |
| FS2 | S | 0,000425 | 0,000585 | S |
| FS3 | S | 0,000317 | 0,000488 | S |
| FS4 | S | 0,000373 | 0,000339 | N |
| FS5 | S | 0,000369 | 0,000476 | S |
| FS6 | S | 0,00042 | 0,000473 | S |
| CM | M | 3,18E-05 | 0,000103 | S |
| CS1 | S | 3,56E-05 | 0,000111 | S |
| CS2 | S | 5,07E-05 | 0,000281 | S |
| CS3 | S | 1,89E-05 | 0,000227 | S |
| CS4 | S | 3,5E-05 | 0,000264 | S |
| EM | M | 0,000139 | 0,000263 | S |
| ES1 | S | 9,12E-05 | 0,000166 | S |
| ES2 | S | 5,43E-05 | 1,48E-05 | N |
| ES3 | S | 6,41E-05 | 3,35E-05 | N |
| ES4 | S | 6,47E-05 | 0,000157 | S |
| ES5 | S | 0,000115 | 0,000221 | S |
| K1M | M | 4,78E-05 | 0,000158 | S |
| K1S1 | S | 9,54E-06 | 3,94E-05 | S |
| K1S2 | S | 1,61E-05 | 5,1E-05 | S |
| K1S3 | S | 1,56E-05 | 0,000179 | S |
| K1S4 | S | 3,53E-05 | 9,36E-06 | N |
| K1S5 | S | 3,84E-06 | 0,000174 | S |
| K2M | M | 0,000112 | 0,000306 | S |
| K2S1 | S | 2,56E-05 | 9,53E-05 | S |
| K2S2 | S | 1,08E-05 | 3,45E-05 | S |
| K2S3 | S | 5,54E-05 | 0,000291 | S |
| K2S4 | S | 9,24E-07 | 1,27E-05 | S |
| K2S5 | S | 3,45E-05 | 0,000221 | S |
| OM | M | 0,000381 | 0,000371 | N |
| OS1 | S | 0,000434 | 0,00034 | N |
| OS2 | S | 0,000451 | 0,000493 | S |
| OS3 | S | 0,000125 | 0,000263 | S |
| OS4 | S | 0,000474 | 0,000364 | N |
| OS5 | S | 0,000123 | 0,000483 | S |
| OS6 | S | 0,000219 | 0,000158 | N |
